# Economic evaluation of HIV pre-exposure prophylaxis strategies: protocol for a methodological systematic review and quantitative synthesis

**DOI:** 10.1186/s13643-018-0710-0

**Published:** 2018-03-15

**Authors:** Kednapa Thavorn, Howsikan Kugathasan, Darrell H. S. Tan, Nasheed Moqueet, Stefan D. Baral, Becky Skidmore, Derek MacFadden, Anna Simkin, Sharmistha Mishra

**Affiliations:** 1 Ottawa Health Research Institute, The Ottawa Hospital, 501 Smyth Road, Ottawa, Ontario K1H 8L6 Canada; 2 School of Epidemiology and Public Health, University of Ottawa, Ottawa, Ontario Canada; 3 Institute for Clinical and Evaluative Sciences, ICES Ottawa, Ottawa, Ontario Canada; 4 Centre for Urban Health Solutions, Li Ka Shing Knowledge Institute, St. Michael’s Hospital, University of Toronto, 209 Victoria Street Rm 315, 3rd Floor, Toronto, Ontario M5B 1T8 Canada; 5 Division of Infectious Diseases, Department of Medicine, University of Toronto, Toronto, Canada; 6 Johns Hopkins School of Public Health, 615 N. Wolfe Street, Baltimore, 21205 USA; 7 Harvard T.H. Chan School of Public Health, Harvard University, 677 Huntington Ave, Boston, 02115 USA; 8 Institute of Health Policy and Management, Dalla Lana School of Public Health, University of Toronto, Toronto, Canada; 9 Institute of Medical Sciences, University of Toronto, Toronto, Canada

**Keywords:** Pre-exposure prophylaxis, Health economic evaluation, Methodological quality, HIV prevention, Mathematical models, HIV transmission models, Cost-effectiveness, Systematic review

## Abstract

**Background:**

Pre-exposure prophylaxis (PrEP) with antiretrovirals is an efficacious and effective intervention to decrease the risk of HIV (human immunodeficiency virus) acquisition. Yet drug and delivery costs prohibit access in many jurisdictions. In the absence of guidelines for the synthesis of economic evaluations, we developed a protocol for a systematic review of economic evaluation studies for PrEP by drawing on best practices in systematic reviews and the conduct and reporting of economic evaluations. We aim to estimate the incremental cost per health outcome of PrEP compared with placebo, no PrEP, or other HIV prevention strategies; assess the methodological variability in, and quality of, economic evaluations of PrEP; estimate the incremental cost per health outcome of different PrEP implementation strategies; and quantify the potential sources of heterogeneity in outcomes.

**Methods:**

We will systematically search electronic databases (MEDLINE, Embase) and the gray literature. We will include economic evaluation studies that assess both costs and health outcomes of PrEP in HIV-uninfected individuals, without restricting language or year of publication. Two reviewers will independently screen studies using predefined inclusion criteria, extract data, and assess methodological quality using the Philips checklist, Second Panel on the Cost-effectiveness of Health and Medicines, and the International Society for Pharmacoeconomics and Outcomes Research recommendations. Outcomes of interest include incremental costs and outcomes in natural units or utilities, cost-effectiveness ratios, and net monetary benefit. We will perform descriptive and quantitative syntheses using sensitivity analyses of outcomes by population subgroups, HIV epidemic settings, study designs, baseline intervention contexts, key parameter inputs and assumptions, type of outcomes, economic perspectives, and willingness to pay values.

**Discussion:**

Findings will guide future economic evaluation of PrEP strategies in terms of methodological and knowledge gaps, and will inform decisions on the efficient integration of PrEP into public health programs across epidemiologic and health system contexts.

**Systematic review registration:**

PROSPERO CRD42016038440.

**Electronic supplementary material:**

The online version of this article (10.1186/s13643-018-0710-0) contains supplementary material, which is available to authorized users.

## Background

In 2016, an estimated 1.8 million people became infected with the human immunodeficiency virus (HIV) [1]. Pre-exposure prophylaxis (PrEP) refers to the use of antiretroviral medications in HIV-uninfected persons to reduce their risk of acquiring HIV and thus reduce population-level transmission [2]. Common PrEP regimens include oral tenofovir (TDF) or a combination of tenofovir and emtricitabine (FTC), and investigation into other agents and formulations is underway [3, 4]. Despite the effectiveness of PrEP, drug and clinical monitoring costs prevent access for many at-risk individuals and limit the scale of PrEP delivery and uptake required to control HIV epidemics [5–9]. Thus, to integrate and deliver PrEP as a scalable component of an HIV prevention program, policy-makers and program implementers must decide about whether, how, and under what conditions resources are best allocated—or reallocated—to PrEP in order to achieve maximum health benefits at affordable costs [6].

Economic evaluations involve a systematic quantification of the trade-offs in costs and consequences of different strategies including single interventions, combination of interventions, or entire programs [10]. Synthesizing outcomes of economic evaluations can be challenging because there are many approaches to the evaluations—each with its strengths and limitations and often driven by data availability, the perspectives under which strategies are evaluated, and the research question and outcomes of interest [11, 12]. However, there are no guidelines for synthesizing the results of economic evaluations.

There exist several recommendations for the conduct and reporting of individual economic evaluations [11, 13–21]. Most recommendations focus on health technology assessments [11, 13–21]—for example, comparison of PrEP regimens. Yet a comparison of PrEP implementation strategies may extend beyond assessing the usual data and modeling decisions needed for health technology assessments. For example, an assessment of PrEP economic evaluations would involve examining whether processes such as the downstream benefits of HIV testing during eligibility screening for PrEP were included in the model [22]; how the fraction of undiagnosed HIV or antiretroviral treatment coverage in persons living with HIV influenced outcomes [23]; how potential ascertainment biases in identifying individuals deemed eligible for PrEP were addressed [24, 25]; and whether the model was calibrated to, and thus reproduced, key epidemiological features of the HIV epidemic pertinent to the strategies evaluated. An example of the latter includes the calibration of high- and lower-risk subgroups in comparisons of PrEP implementation strategies prioritized to subgroups at higher risk of HIV.

The methods, inputs, and assumptions in existing PrEP economic evaluations and their influence on outcomes have yet to be assessed with a systematic review. One systematic review summarized findings from 13 economic evaluations of PrEP that used transmission dynamic models published before 2013 [26]. The review provided a detailed and narrative synthesis of each study stratified by epidemic context (high-prevalence generalized epidemics vs. lower prevalence concentrated epidemics) and noted that PrEP strategies prioritized to key populations (such as men who have sex with men) with the highest risk of HIV exposure were most cost-effective [26]. However, the review was restricted to transmission dynamic models and did not describe the methodological strengths and limitations nor how assumptions may have influenced the results [26]. Another systematic review of pre-2014 studies identified only two economic evaluations and briefly summarized their main results [27]. In 2016, an expert review described in narrative detail the methods and findings from the seven most recent (published after 2014) cost-effectiveness studies of introducing PrEP [28]. The findings were stratified by geography, and authors outlined an in-depth discussion of how model parameters may influence outcomes—noting that conditions such as high baseline HIV incidence and PrEP adherence led to cost savings [28]. The expert review also identified key gaps, including a need to understand how real-world PrEP implementation factors influence cost-effectiveness and budgetary impact [28]. However, the two reviews did not examine every single scenario simulated in each primary study nor the various perspectives and funding systems for PrEP. Thus, the reviews could not quantitatively explore potential sources of variability in the projected outcomes [26, 28].

We aim to estimate the cost-effectiveness of all PrEP and PrEP implementation strategies evaluated to date in the published literature and systematically appraise and quantify the methodological and other sources of heterogeneity in outcomes. In the absence of guidelines for the synthesis of economic evaluations, we developed a protocol for a systematic review of economic evaluation studies for PrEP by drawing on best practices in systematic reviews and the conduct and reporting of economic evaluations.

## Methods

### Study design

We will conduct a systematic review of economic evaluation studies comparing PrEP to placebo, the status quo (no placebo, no PrEP), or other HIV prevention interventions and studies comparing different types of PrEP implementation strategies. The protocol is registered in the PROSPERO database (CRD42016038440) and was prepared in accordance with the Preferred Reporting Items for Systematic Reviews and Meta-analyses statement for protocols [29] (Additional file 1).

Objectives of the systematic review:

1. To estimate the incremental cost per health outcome of PrEP compared with placebo, status quo (no placebo, no PrEP), or other HIV prevention strategies.
2. To assess the methodological variability in, and quality of, economic evaluations of PrEP.
3. To estimate the incremental cost per health outcome of different types of PrEP implementation strategies.
4. To quantify the potential sources of heterogeneity (data, assumptions, methodological) in the cost-effectiveness of PrEP implementation strategies.

### Primary outcomes

Our primary outcomes are incremental cost-effectiveness ratios, such as incremental cost per HIV infection averted; incremental cost per death averted; incremental cost per life year saved, or quality-adjusted life years (QALY), or disability-adjusted life year (DALY); and incremental net monetary benefit.

### Information sources and search strategies

An experienced information specialist will produce and test the preliminary electronic search strategies using an iterative process in collaboration with the research team. Using the OVID platform, an economic literature search will be performed in Ovid MEDLINE® Epub Ahead of Print, In-Process & Other Non-Indexed Citations, and Ovid MEDLINE® and Embase Classic+Embase. NHS Economic Evaluation Database in the Cochrane Library (Wiley version) will also be searched. We will evaluate the search strategy by ensuring it yields all of the primary studies identified from the three previously published systematic reviews [26–28]. Additional file 2 details the complete search strategy. We will also conduct a gray literature search using websites of relevant health technology assessment organizations and national-level agencies provided by the Health Technology Assessment international (HTAi, Additional file 2).

### Eligibility criteria for considering studies for the review

We will include economic evaluations of PrEP provided to individuals at risk of HIV acquisition through sex or needle exchange. PrEP regimens may involve any antiretroviral drug (e.g., tenofovir, tenofovir/emtricitabine, rilpivirine, dapivirine). The regimens can be at any dose, formulation (e.g., tablet, topical vaginal/anal gel, long-lasting injection, intravaginal ring), and duration (e.g., intermittent, fixed duration, lifetime use). Studies may evaluate the use of PrEP alone, or in combination with other HIV prevention strategies, and any PrEP implementation strategy (focused on specific subpopulations, scaled roll-out over time, adherence-support programs, etc.).

Eligible study designs comprise full economic evaluations comparing both costs and outcomes of PrEP including cost-minimization analyses, cost-benefit analyses, cost-effectiveness analyses, or cost-utility analyses, without restriction on the language of publication or year of study.

### Target population

We will restrict economic evaluations where the intervention (PreP) is provided to, and outcomes are estimated among, individuals age 14 years and above (including pregnant or breastfeeding women) and without geographical restriction.

### Types of studies to be excluded

We will exclude editorials, conference abstracts, and economic evaluations focused solely on parent-to-child transmission of HIV.

### Screening and selection

Citations will be de-duplicated and managed in Covidence® (Melbourne, Australia). We will perform study selection in two stages. In stage 1, two reviewers will independently screen the titles and abstracts for eligibility. To minimize errors and maximize the efficiency of the review process, we will pilot a random sample of 50 citations to identify those that meet eligibility criteria and calculate the inter-rater agreement for study inclusion and proceed with a dual review after > 90% agreement is achieved with the same reviewers. Upon completion of the dual screening, we will calculate the inter-rater agreement and resolve discrepancies through a third reviewer. Stage 2 will include a similar process for screening the full text of articles. Co-publications or multiple reports of the same study will be identified as such.

### Data abstraction and data collection process

Two reviewers will independently perform data extraction from included studies using a standardized form (Additional file 3) developed in an online database (Sonadier, Sonaider Inc. https://www.sonadier.com/), with inter-rater agreement calculated and disagreements resolved by discussion. The two reviewers will first pilot a random sample of five studies and begin final abstraction after inter-reviewer agreement exceeds 90% in the pilot. Extracted data will include study characteristics, baseline HIV epidemic characteristics (HIV prevalence, existing HIV interventions), population characteristics (HIV risk factors, co-morbidities), PrEP regimens (antiretroviral agents, formulations, route of administration, dose/duration/frequency of use), implementation strategy (prioritization, uptake/coverage, adherence), and comparator(s). We will record the type of economic evaluation (e.g., cost minimization, cost-benefit, cost-effectiveness, or cost-utility analysis), study setting and currency, willingness-to-pay threshold, modeling method (e.g., decision analytic, cohort, microsimulation, transmission dynamic), perspective of analysis (patient, hospital, health care system, or societal), cost components and details (direct medical cost, direct non-medical cost, and indirect cost), health benefits (monetary term, natural unit, or quality-adjusted life years), discount rates, approach to sensitivity analyses (deterministic, probabilistic), and reporting of results (efficiency frontiers, etc.).

We will extract outcomes reported in the primary studies: incremental cost, incremental outcomes (e.g., HIV infection averted, death averted, life-year saved, quality-adjusted life years [QALY], or disability-adjusted life year [DALY]), incremental cost-effectiveness ratios (e.g., incremental cost per HIV infection averted, incremental cost per death averted, incremental cost per QALY gained), or incremental net benefit. We will extract discounted and undiscounted values as reported. In cases where an incremental cost-effectiveness ratio is not explicitly provided in included studies, we will derive an incremental cost-effectiveness ratio from the reported findings. We will extract data on every scenario examined within a study, including sensitivity analyses. If results of sensitivity analyses are only available in figures, study authors will be contacted to request the numerical values.

We will convert cost data to 2017 US dollars (USD) for comparison across study settings using annual exchange rates and consumer price index reported by the US Bureau of Labor Statistics [30]. The online database will be made open-access upon publication of the findings and will readily support future updates to the search and data extraction.

### Methods appraisal

We will combine established tools to appraise the economic evaluations (Additional file 4). We will assess the quality of reporting of health economic evaluation studies using the CHEERS (Consolidated Health Economic Evaluation Reporting Standards) checklist [15] and the Second Panel on Cost-effectiveness in Health and Medicine [14]. We will use the Philips checklist [11] to appraise the three domains that govern model quality in economic models: structure, data inputs, and consistency (Additional file 4). Assessment of model structure covers the following: a clearly stated decision problem, objectives, rationale, interventions/comparators, model type (selection and justification), time horizon, disease pathways, and cycle length. The data input domain examines how input parameters are described and justified, and whether various types of uncertainties are adequately assessed and reported. The consistency domain examines whether the economic model and its results are compared with the results from other models. We will supplement the Phillips checklist with the good research practice recommendations from the International Society for Pharmacoeconomics and Outcomes Research (ISPOR) for decision analytic, cohort, discrete event, and transmission dynamics models [16–21]. The additional recommendations cover a fourth domain: model calibration and structural model assumptions such as heterogeneity in HIV acquisition risk and sexual mixing patterns, which can influence outcomes of prioritization strategies [31].

Two team members (KT and SM) will independently appraise study methods across the four domains, using a categorical scale, and resolve conflicts through discussion and consensus. Results from the method assessment and the implications for generalizability (akin to the risk of bias assessment) will be presented in table format with shading for easy visualization.

### Synthesis: narrative and quantitative

#### Narrative summary and identifying knowledge gaps

We will describe the median and range of outcomes overall and by the following:

- Study characteristics: study perspective and setting (low-middle income vs. high-income country, gross domestic product per capita, health care expenditure per capita)
- Data inputs and assumptions: baseline HIV epidemic (pre-intervention HIV incidence and prevalence), baseline intervention context (antiretroviral treatment coverage), and intervention costs (e.g., cost per individual on PrEP)
- Methods: type of economic evaluation, modeling method, model structure (e.g., sexual and/or injecting partnership and network structure), discounting rate, and health outcomes measure and instruments
- PrEP intervention and implementation strategies: PrEP regimen, population subgroups who receive PrEP (by age, sex, gender identify, sexual orientation, ethnicity); coverage/uptake; and adherence. Population subgroups need not be mutually exclusive and include gay, bisexual, and other men who have sex with men; sex workers; persons who inject drugs; serodiscordant partnerships; and transgender persons.

To identify knowledge gaps, we will create a matrix with elements that count the number of studies and modeled scenarios that evaluated each PrEP regimen and each implementation strategy, stratified by study characteristics, data inputs and assumptions, methods, and by population subgroups who receive PrEP. The elements within the matrix will thus provide information about what has not been evaluated—and under which epidemic contexts—using which methods and in which subgroups. We will include all possible combinations of PrEP regimens and implementation strategies in the matrix.

#### Quantitative synthesis

First, we will display the one-way sensitivity analyses using scatter plots and tornado plots to identify sources of heterogeneity from each of the above categories (study characteristics, data inputs and assumptions, methods, and PrEP intervention and implementation strategies). For outcomes of the same type and with the same comparator (e.g., comparison of a PrEP intervention with no PrEP), we will then explore one-way correlations of outcomes with the above potential sources of heterogeneity using partial rank correlation coefficients [31]. Variables with the largest correlation coefficients represent the largest source of variability in outcomes.

### Dissemination

To assure the broad dissemination and applications of our study findings, our knowledge translation activities will include peer-reviewed publications and presentations at scientific meetings and a one-page evidence brief and infographic for wider dissemination via social media. The quantitative sensitivity analyses will be reproduced as a graphical user interface with R Shiny (open access software, https://shiny.rstudio.com/) so that knowledge users can interactively use the platform to identify which parameters (or methods) produce the outcomes observed from the primary studies. The link to the website and platform will be open access and publicly accessible as part of the final peer-reviewed manuscript, and with links to the infographic and evidence brief.

## Discussion

Economic evaluations are important for healthcare policy decisions in an era of financial constraints. Thus, policy decisions could be best informed by a clear understanding of how heterogeneity in methods, data and structural assumptions, and implementation conditions influence the outcome of interest. In the case of HIV prevention, few systematic reviews of economic evaluations have been conducted [26], and even fewer have attempted to quantify potential sources of variability in the reported outcomes [26]. We developed a protocol for a systematic review of economic evaluations of HIV PrEP strategies that aims to assess the methodological quality of economic evaluations of PrEP, quantify sources of variability in projected cost-effectiveness results, and identify key knowledge gaps.

By 2016, two of eight clinical guidelines and position statements for PrEP mentioned the importance of considering cost when generating recommendations [32, 33]. As guidelines begin to utilize economic evaluations [28, 34], a more nuanced and quantitative synthesis of the sources of variability in cost-effectiveness ratios could better equip decision-makers to determine the generalizability of existing economic evaluations to their local context or to call for specific economic evaluations to meet their needs. To aid this process, our dissemination platform will enable end-users to intuit the key implications of the findings by selecting the features that are relevant to their needs. For example, some policy-makers may only wish to use estimates that capture onward transmission (i.e., methods that used transmission dynamic models), or health providers may wish to draw upon estimates based on high PrEP adherence if that is most relevant to their patient population. However, our synthesis will not include an assessment of the strength of the evidence as the process includes a comprehensive and joint review of the empirical (i.e., effectiveness) and modeled evidence (e.g., cost-effectiveness) and is best suited to clinical and public health guidelines [28, 34].

Systematic reviews of economic evaluation and of mathematical modeling studies are emerging in the published literature [35]. However, we identified several challenges in developing a high-quality systematic review and quantitative synthesis of economic evaluations. In the absence of recommendations for the synthesis of economic evaluations, there remains a lack of consensus surrounding the best instrument for assessing the quality of economic evaluation as part of a systematic review—especially given the heterogeneity in the types of models that can be appropriately and justifiably used. To address this gap, we will leverage CHEERS [15], the Second Panel of Cost-effectiveness in Health and Medicine [14], the Phillips checklist [11], and the ISPOR recommendations for health economic evaluations using various types of models, including transmission dynamic models [16–21]. Second, the prior reviews suggest substantial heterogeneity in clinical assumptions and methodological features across studies and PrEP interventions [26, 28]. We will turn the challenge into an opportunity by performing a quantitative sensitivity analyses that stratify outcomes according to variables anticipated to influence cost-effectiveness, such as subgroups, baseline epidemic context, study designs, and type of outcomes. Formal meta-analyses using measures of statistical heterogeneity are not validated for use in outcomes projected from simulation models. Thus, we will apply traditional, non-parametric approaches to sensitivity analyses developed for within-model examination and to our between-model analyses [31]. Understanding the sources of variability in cost-effectiveness outcomes will provide a more robust basis for decision-making and generalizability.

Deliberations regarding public, governmental, or insurance provider funding for PrEP are currently underway in several countries [36–40]. Findings from this systematic review can be used across jurisdictions to inform decisions on adoption and integration of PrEP implementation strategies into public health programs across epidemiologic and health system contexts. The review can also help identify key knowledge gaps to help prioritize future modeling studies, ensuring that modeling efforts are themselves optimized to generate new knowledge and to meet the needs of decision-makers, and in turn, to inform the effective and efficient delivery of PrEP as part of the HIV prevention response.

## Additional files

Additional file 1: PRISMA-P checklist. (DOCX 36 kb)

Additional file 2: Search strategies. (DOCX 36 kb)

Additional file 3: Data abstraction form. (DOCX 41 kb)

Additional file 4: Quality assessment. (DOCX 34 kb)

## Abbreviations

ART: Antiretroviral treatment
CHEERS: Consolidated Health Economic Evaluation Reporting Standards
DALY: Disability-adjusted life year
FTC: Emtricitabine
HIV: Human immunodeficiency virus
HTAi: Health Technology Assessment international
ISPOR: International Society for Pharmacoeconomics and Outcomes Research
PrEP: Pre-exposure prophylaxis
QALY: Quality-adjusted life year
TDF: Tenofovir
USD: US dollars
