# Supplementary material for: Economic evaluation of HIV pre-exposure prophylaxis strategies: protocol for a methodological systematic review and quantitative synthesis
Source: Syst Rev. 2018 Mar 15;7:47. doi: 10.1186/s13643-018-0710-0 (PMC5855998; doi:10.1186/s13643-018-0710-0)
Supplement: Supplementary file 2 — Search strategies. (DOCX 36 kb) [file 13643_2018_710_MOESM2_ESM.docx]

**Additional File 2**

**Search strategies and grey literature search**

MEDLINE

Database: Ovid MEDLINE(R) In-Process & Other Non-Indexed Citations and Ovid MEDLINE(R) <1946 to Present>

Search Strategy:

--------------------------------------------------------------------------------

1 HIV Seronegativity/ (3421)

2 ((AIDs or (acquired immune deficiency* adj2 virus) or (acquired immunodeficiency* adj2 virus*) or human immunodeficiency virus* or human immuno-deficiency virus*) adj2 (negativ* or seronegativ* or sero-negativ*)).tw,kw. (1080)

3 ((HIV or HIV-1 or HIV1 or HIV-I or HIVI or HIV-2 or HIV2 or HIV-2 or HIV-II or HIVII or HTLV* or LAV-2 or LAV-II) adj2 (negativ* or seronegativ* or sero-negativ*)).tw,kw. (13216)

4 ((uninfected or "not infected") adj2 (HIV or HIV-1 or HIV1 or HIV-I or HIVI or HIV-2 or HIV2 or HIV-2 or HIV-II or HIVII or HTLV* or LAV-2 or LAV-II)).tw,kw. (3006)

5 ((uninfected or "not infected") adj2 (AIDs or (acquired immune deficiency* adj2 virus) or (acquired immunodeficiency* adj2 virus*) or human immunodeficiency virus* or human immuno-deficiency virus*)).tw,kw. (155)

6 (without adj (HIV or HIV-1 or HIV1 or HIV-I or HIVI or HIV-2 or HIV2 or HIV-2 or HIV-II or HIVII or HTLV* or LAV-2 or LAV-II)).tw,kw. (1438)

7 (without adj (AIDs or (acquired immune deficiency* adj2 virus) or (acquired immunodeficiency* adj2 virus*) or human immunodeficiency virus* or human immuno-deficiency virus*)).tw,kw. (965)

8 ((non or "not") adj (HIV or HIV-1 or HIV1 or HIV-I or HIVI or HIV-2 or HIV2 or HIV-2 or HIV-II or HIVII or HTLV* or LAV-2 or LAV-II) adj positiv*).tw,kw. (86)

9 (non-positiv* adj (HIV or HIV-1 or HIV1 or HIV-I or HIVI or HIV-2 or HIV2 or HIV-2 or HIV-II or HIVII or HTLV* or LAV-2 or LAV-II)).tw,kw. (0)

10 ((non or "not") adj (AIDs or (acquired immune deficiency* adj2 virus) or (acquired immunodeficiency* adj2 virus*) or human immunodeficiency virus* or human immuno-deficiency virus*) adj positiv*).tw,kw. (1)

11 (non-positiv* adj (AIDs or (acquired immune deficiency* adj2 virus) or (acquired immunodeficiency* adj2 virus*) or human immunodeficiency virus* or human immuno-deficiency virus*)).tw,kw. (0)

12 or/1-11 (19559)

13 exp HIV Infections/pc [Prevention & Control] (43090)

14 (prevent* adj3 (AIDs or (acquired immune deficiency* adj2 virus) or (acquired immunodeficiency* adj2 virus*) or human immunodeficiency virus* or human immuno-deficiency virus*)).tw,kw. (5158)

15 (prevent* adj3 (HIV or HIV-1 or HIV1 or HIV-I or HIVI or HIV-2 or HIV2 or HIV-2 or HIV-II or HIVII or HTLV* or LAV-2 or LAV-II)).tw,kw. (17166)

16 (prophyla* adj3 (AIDs or (acquired immune deficiency* adj2 virus) or (acquired immunodeficiency* adj2 virus*) or human immunodeficiency virus* or human immuno-deficiency virus*)).tw,kw. (329)

17 (prophyla* adj3 (HIV or HIV-1 or HIV1 or HIV-I or HIVI or HIV-2 or HIV2 or HIV-2 or HIV-II or HIVII or HTLV* or LAV-2 or LAV-II)).tw,kw. (1519)

18 or/13-17 (52429)

19 Anti-Retroviral Agents/ (6934)

20 Anti-HIV Agents/ (35526)

21 (antiretrovir* or anti-retrovir* or antiHIV* or anti-HIV* or antiAIDS or anti-AIDS).tw,kw. (58506)

22 Adenine/aa (7899)

23 tenofovir*.tw,kw. (3986)

24 107021-12-5.rn. (0)

25 (rilpivirine or edurant or TMC-278 or TMC278 or UNII-FI96A8X663).tw,kw. (350)

26 rilpivirine.rn. (184)

27 (dapivirine or TMC-120 or TMC120 or UNII-TCN4MG2VXS).tw,kw. (106)

28 dapivirine.rn. (0)

29 Truvada.tw,kw. (110)

30 ("TDF-FTC" or "FTC-TDF" or "TDF/FTC" or "FTC/TDF").tw,kw. (353)

31 731772-45-5.rn. (0)

32 or/19-31 (82816)

33 Primary Prevention/ or Pre-Exposure Prophylaxis/ (15617)

34 32 and 33 (364)

35 12 and (18 or 34) (2414)

36 18 and 32 (8911)

37 exp HIV Infections/ (243267)

38 exp HIV/ (86554)

39 HIV Seroprevalence/ (3079)

40 (AIDs or (acquired immune deficiency* adj2 virus) or (acquired immunodeficiency* adj2 virus*) or human immunodeficiency virus* or human immuno-deficiency virus*).tw,kw. (190207)

41 (HIV or HIV-1 or HIV1 or HIV-I or HIVI or HIV-2 or HIV2 or HIV-2 or HIV-II or HIVII or HTLV* or LAV-2 or LAV-II).tw,kw. (261776)

42 or/37-41 (377117)

43 Pre-Exposure Prophylaxis/ (303)

44 ((pre-expos* or preexpos*) adj3 prophyla*).tw,kw. (1211)

45 ((pre-expos* or preexpos*) adj3 protect*).tw,kw. (71)

46 PrEP.tw,kw. (2315)

47 or/43-46 (3086)

48 (1 or 42) and 47 (1182)

49 35 or 36 or 48 (10977)

50 Economics/ (26664)

51 exp "Costs and Cost Analysis"/ (195358)

52 Economics, Nursing/ (3933)

53 Economics, Medical/ (8858)

54 Economics, Pharmaceutical/ (2608)

55 exp Economics, Hospital/ (21229)

56 Economics, Dental/ (1876)

57 exp "Fees and Charges"/ (28073)

58 exp Budgets/ (12765)

59 budget*.ti,ab. (21622)

60 (economic* or cost or costs or costly or costing or price or prices or pricing or pharmacoeconomic* or pharmaco-economic* or expenditure or expenditures or expense or expenses or financial or finance or finances or financed).ti. (143724)

61 (economic* or cost or costs or costly or costing or price or prices or pricing or pharmacoeconomic* or pharmaco-economic* or expenditure or expenditures or expense or expenses or financial or finance or finances or financed).ab. /freq=2 (197230)

62 (cost* adj2 (effective* or utilit* or benefit* or minimi* or analy* or outcome or outcomes)).ab. (108287)

63 (value adj2 (money or monetary)).ti,ab. (1577)

64 exp models, economic/ (11410)

65 Quality-Adjusted Life Years/ (8146)

66 (life qualities or life quality or quality adjusted or adjusted life or qol or qoly or qolys or hrqol or qaly or qalys or qale or qales).tw. (47113)

67 exp Models, Economic/ (11410)

68 economic model*.ti,ab. (2145)

69 markov chains/ (10984)

70 markov.ti,ab. (14490)

71 monte carlo method/ (22213)

72 monte carlo.ti,ab. (33870)

73 exp Decision Theory/ (10235)

74 (decision* adj2 (tree* or analy* or model*)).ti,ab. (15159)

75 or/50-74 (576882)

76 49 and 75 (884)

77 "Quality of Life"/ (134411)

78 quality-adjusted life years/ (8146)

79 (life adj1 (quality or qualities)).ti,ab. (4728)

80 (adjusted adj1 (quality or life)).ti,ab. (10271)

81 (qol or qoly or qolys or hrqol or qaly or qalys or qale or qales).ti,ab. (38560)

82 or/77-81 (155281)

83 49 and 82 (156)

84 76 or 83 (912)

85 exp Animals/ not (exp Animals/ and Humans/) (4208134)

86 84 not 85 (908)

***************************

Embase

Database: Embase Classic+Embase <1947 to 2016 April 05>

Search Strategy:

--------------------------------------------------------------------------------

1 ((AIDs or (acquired immune deficiency* adj2 virus) or (acquired immunodeficiency* adj2 virus*) or human immunodeficiency virus* or human immuno-deficiency virus*) adj2 (negativ* or seronegativ* or sero-negativ*)).tw,kw. (1139)

2 ((HIV or HIV-1 or HIV1 or HIV-I or HIVI or HIV-2 or HIV2 or HIV-2 or HIV-II or HIVII or HTLV* or LAV-2 or LAV-II) adj2 (negativ* or seronegativ* or sero-negativ*)).tw,kw. (16609)

3 ((uninfected or "not infected") adj2 (HIV or HIV-1 or HIV1 or HIV-I or HIVI or HIV-2 or HIV2 or HIV-2 or HIV-II or HIVII or HTLV* or LAV-2 or LAV-II)).tw,kw. (3800)

4 ((uninfected or "not infected") adj2 (AIDs or (acquired immune deficiency* adj2 virus) or (acquired immunodeficiency* adj2 virus*) or human immunodeficiency virus* or human immuno-deficiency virus*)).tw,kw. (155)

5 (without adj (HIV or HIV-1 or HIV1 or HIV-I or HIVI or HIV-2 or HIV2 or HIV-2 or HIV-II or HIVII or HTLV* or LAV-2 or LAV-II)).tw,kw. (1902)

6 (without adj (AIDs or (acquired immune deficiency* adj2 virus) or (acquired immunodeficiency* adj2 virus*) or human immunodeficiency virus* or human immuno-deficiency virus*)).tw,kw. (1094)

7 ((non or "not") adj (HIV or HIV-1 or HIV1 or HIV-I or HIVI or HIV-2 or HIV2 or HIV-2 or HIV-II or HIVII or HTLV* or LAV-2 or LAV-II) adj positiv*).tw,kw. (100)

8 (non-positiv* adj (HIV or HIV-1 or HIV1 or HIV-I or HIVI or HIV-2 or HIV2 or HIV-2 or HIV-II or HIVII or HTLV* or LAV-2 or LAV-II)).tw,kw. (0)

9 ((non or "not") adj (AIDs or (acquired immune deficiency* adj2 virus) or (acquired immunodeficiency* adj2 virus*) or human immunodeficiency virus* or human immuno-deficiency virus*) adj positiv*).tw,kw. (1)

10 (non-positiv* adj (AIDs or (acquired immune deficiency* adj2 virus) or (acquired immunodeficiency* adj2 virus*) or human immunodeficiency virus* or human immuno-deficiency virus*)).tw,kw. (0)

11 or/1-10 (23038)

12 exp Human immunodeficiency virus infection/pc [Prevention] (41705)

13 (prevent* adj3 (AIDs or (acquired immune deficiency* adj2 virus) or (acquired immunodeficiency* adj2 virus*) or human immunodeficiency virus* or human immuno-deficiency virus*)).tw,kw. (5147)

14 (prevent* adj3 (HIV or HIV-1 or HIV1 or HIV-I or HIVI or HIV-2 or HIV2 or HIV-2 or HIV-II or HIVII or HTLV* or LAV-2 or LAV-II)).tw,kw. (19920)

15 (prophyla* adj3 (AIDs or (acquired immune deficiency* adj2 virus) or (acquired immunodeficiency* adj2 virus*) or human immunodeficiency virus* or human immuno-deficiency virus*)).tw,kw. (363)

16 (prophyla* adj3 (HIV or HIV-1 or HIV1 or HIV-I or HIVI or HIV-2 or HIV2 or HIV-2 or HIV-II or HIVII or HTLV* or LAV-2 or LAV-II)).tw,kw. (1963)

17 or/12-16 (54443)

18 antiretrovirus agent/ (36159)

19 anti human immunodeficiency virus agent/ (15844)

20 (antiretrovir* or anti-retrovir* or antiHIV* or anti-HIV* or antiAIDS or anti-AIDS).tw,kw. (74822)

21 tenofovir/ (12762)

22 tenofovir.rn. (10558)

23 rilpivirine/ (1197)

24 rilpivirine.rn. (998)

25 dapivirine/ (402)

26 dapivirine.rn. (33)

27 emtricitabine plus tenofovir disoproxil/ (2565)

28 Truvada.tw,kw. (1184)

29 ("TDF-FTC" or "FTC-TDF" or "TDF/FTC" or "FTC/TDF").tw,kw. (870)

30 731772-45-5.rn. (0)

31 or/18-30 (104517)

32 prophylaxis/ or pre-exposure prophylaxis/ (91978)

33 31 and 32 (2161)

34 11 and (17 or 33) (2436)

35 17 and 31 (11065)

36 exp Human immunodeficiency virus infection/ (322713)

37 exp Human immunodeficiency virus/ (156368)

38 human immunodeficiency virus prevalence/ (8057)

39 (AIDs or (acquired immune deficiency* adj2 virus) or (acquired immunodeficiency* adj2 virus*) or human immunodeficiency virus* or human immuno-deficiency virus*).tw,kw. (224360)

40 (HIV or HIV-1 or HIV1 or HIV-I or HIVI or HIV-2 or HIV2 or HIV-2 or HIV-II or HIVII or HTLV* or LAV-2 or LAV-II).tw,kw. (322657)

41 or/36-40 (493380)

42 pre-exposure prophylaxis/ (664)

43 ((pre-expos* or preexpos*) adj3 prophyla*).tw,kw. (1622)

44 ((pre-expos* or preexpos*) adj3 protect*).tw,kw. (82)

45 PrEP.tw,kw. (4573)

46 or/42-45 (5559)

47 41 and 46 (1692)

48 34 or 35 or 47 (13315)

49 economics/ (222046)

50 exp cost/ (285788)

51 exp health economics/ (688359)

52 exp fee/ (36525)

53 budget/ (22637)

54 budget*.ti,ab. (28643)

55 (economic* or cost or costs or costly or costing or price or prices or pricing or pharmacoeconomic* or pharmaco-economic* or expenditure or expenditures or expense or expenses or financial or finance or finances or financed).ti. (187536)

56 (economic* or cost or costs or costly or costing or price or prices or pricing or pharmacoeconomic* or pharmaco-economic* or expenditure or expenditures or expense or expenses or financial or finance or finances or financed).ab. /freq=2 (274166)

57 (cost* adj2 (effective* or utilit* or benefit* or minimi* or analy* or outcome or outcomes)).ab. (148946)

58 (value adj2 (money or monetary)).ti,ab. (2230)

59 statistical model/ (121818)

60 economic model*.ti,ab. (3190)

61 probability/ (64196)

62 markov.ti,ab. (18421)

63 monte carlo method/ (26700)

64 monte carlo.ti,ab. (32727)

65 decision theory/ (1609)

66 (decision* adj2 (tree* or analy* or model*)).ti,ab. (20803)

67 or/49-66 (1248651)

68 48 and 67 (2162)

69 exp "quality of life"/ (333100)

70 (life adj1 (quality or qualities)).ti,ab. (8731)

71 (adjusted adj1 (quality or life)).ti,ab. (14649)

72 (qol or qoly or qolys or hrqol or qaly or qalys or qale or qales).ti,ab. (68146)

73 or/69-72 (345349)

74 48 and 73 (332)

75 68 or 74 (2303)

76 exp animal experimentation/ or exp models animal/ or exp animal experiment/ or nonhuman/ or exp vertebrate/ (22942727)

77 exp human/ or exp human experimentation/ or exp human experiment/ (17160227)

78 76 not 77 (5783525)

79 75 not 78 (2293)

80 conference abstract.pt. (2192127)

81 79 not 80 (2158)

***************************

Cochrane Library

Search Name: HIV Pre-Exposure Prophylaxis (PrEP)

Date Run: 06/04/16 15:24:55.984

Description: OHRI (Kednapa) - 2015 Dec 1

ID Search Hits

#1 [mh "HIV Seropositivity"] Publication Year from 1988 to 1993 77

#2 [mh "HIV Seronegativity"] 157

#3 ((AIDS or ("acquired immune deficiency" near/2 virus*) or ("acquired immunodeficiency" near/2 virus*) or ("human immunodeficiency" next virus*) or ("human immuno-deficiency" next virus*)) near/2 (negativ* or seronegativ* or sero-negativ*)):ti,ab,kw 68

#4 ((HIV or "HIV-1" or HIV1 or "HIV-I" or HIVI or "HIV-2" or HIV2 or "HIV-2" or "HIV-II" or HIVII or HTLV* or "LAV-2" or "LAV-II") near/2 (negativ* or seronegativ* or (sero next negativ*))):ti,ab,kw 864

#5 ((uninfected or "not infected") near/2 (HIV or "HIV-1" or HIV1 or "HIV-I" or HIVI or "HIV-2" or HIV2 or "HIV-2" or "HIV-II" or HIVII or HTLV* or "LAV-2" or "LAV-II")):ti,ab,kw 409

#6 ((uninfected or "not infected") near/2 (AIDS or ("acquired immune deficiency" near/2 virus*) or ("acquired immunodeficiency" near/2 virus*) or ("human immunodeficiency" next virus*) or ("human immuno-deficiency" next virus*))):ti,ab,kw 20

#7 (without next (HIV or "HIV-1" or HIV1 or "HIV-I" or HIVI or "HIV-2" or HIV2 or "HIV-2" or "HIV-II" or HIVII or HTLV* or "LAV-2" or "LAV-II")):ti,ab,kw 71

#8 (without next (AIDS or ("acquired immune deficiency" near/2 virus*) or ("acquired immunodeficiency" near/2 virus*) or ("human immunodeficiency" next virus*) or ("human immuno-deficiency" next virus*))):ti,ab,kw 46

#9 ((non or "not") next (HIV or "HIV-1" or HIV1 or "HIV-I" or HIVI or "HIV-2" or HIV2 or "HIV-2" or "HIV-II" or HIVII or HTLV* or "LAV-2" or "LAV-II") next positiv*):ti,ab,kw 5

#10 ((non next positiv*) next (HIV or "HIV-1" or HIV1 or "HIV-I" or HIVI or "HIV-2" or HIV2 or "HIV-2" or "HIV-II" or HIVII or HTLV* or "LAV-2" or "LAV-II")):ti,ab,kw 0

#11 ((non or "not") next (AIDS or ("acquired immune deficiency" near/2 virus*) or ("acquired immunodeficiency" near/2 virus*) or ("human immunodeficiency" next virus*) or ("human immuno-deficiency" next virus*)) next positiv*):ti,ab,kw 0

#12 ((non next positiv*) next (AIDS or ("acquired immune deficiency" near/2 virus*) or ("acquired immunodeficiency" near/2 virus*) or ("human immunodeficiency" next virus*) or ("human immuno-deficiency" next virus*))):ti,ab,kw 0

#13 {or #1-#12} 1407

#14 [mh "HIV Infections"/PC] 2145

#15 (prevent* near/3 (AIDS or ("acquired immune deficiency" near/2 virus*) or ("acquired immunodeficiency" near/2 virus*) or ("human immunodeficiency" next virus*) or ("human immuno-deficiency" next virus*))):ti,ab,kw 675

#16 (prevent* near/3 (HIV or "HIV-1" or HIV1 or "HIV-I" or HIVI or "HIV-2" or HIV2 or "HIV-2" or "HIV-II" or HIVII or HTLV* or "LAV-2" or "LAV-II")):ti,ab,kw 1744

#17 (prophyla* near/3 (AIDS or ("acquired immune deficiency" near/2 virus*) or ("acquired immunodeficiency" near/2 virus*) or ("human immunodeficiency" next virus*) or ("human immuno-deficiency" next virus*))):ti,ab,kw 43

#18 (prophyla* near/3 (HIV or "HIV-1" or HIV1 or "HIV-I" or HIVI or "HIV-2" or HIV2 or "HIV-2" or "HIV-II" or HIVII or HTLV* or "LAV-2" or "LAV-II")):ti,ab,kw 197

#19 {or #14-#18} 3205

#20 [mh ^"Anti-Retroviral Agents"] 486

#21 [mh ^"Anti-HIV Agents"] 2456

#22 (antiretrovir* or (anti next retrovir*) or antiHIV* or (anti next HIV*) or antiAIDS or (anti next AIDS)):ti,ab,kw 5716

#23 [mh Adenine/AA] 603

#24 tenofovir*:ti,ab,kw 1138

#25 (rilpivirine or edurant or "TMC-278" or TMC278 or "UNII-FI96A8X663"):ti,ab,kw 99

#26 (dapivirine or "TMC-120" or TMC120 or "UNII-TCN4MG2VXS"):ti,ab,kw 11

#27 Truvada:ti,ab,kw 17

#28 ("TDF-FTC" or "FTC-TDF" or "TDF/FTC" or "FTC/TDF"):ti,ab,kw 274

#29 {or #20-#28} 6392

#30 [mh ^"Primary Prevention"] or [mh "Pre-Exposure Prophylaxis"] 958

#31 #29 and #30 27

#32 #13 and (#19 or #31) 451

#33 #19 and #29 742

#34 [mh "HIV Infections"] 8933

#35 [mh HIV] 2824

#36 [mh "HIV Seroprevalence"] 43

#37 (AIDS or ("acquired immune deficiency" near/2 virus*) or ("acquired immunodeficiency" near/2 virus*) or ("human immunodeficiency" next virus*) or ("human immuno-deficiency" next virus*)):ti,ab,kw 10045

#38 (HIV or "HIV-1" or HIV1 or "HIV-I" or HIVI or "HIV-2" or HIV2 or "HIV-2" or "HIV-II" or HIVII or HTLV* or "LAV-2" or "LAV-II"):ti,ab,kw 13409

#39 {or #34-#38} 16585

#40 [mh "Pre-Exposure Prophylaxis"] 25

#41 (((pre next expos*) or preexpos*) near/3 prophyla*):ti,ab,kw 182

#42 (((pre next expos*) or preexpos*) near/3 prevent*):ti,ab,kw 10

#43 PrEP:ti,ab,kw 320

#44 {or #40-#43} 406

#45 (#1 or #2 or #39) and #44 165

#46 #32 or #33 or #45 1071

DSR – 31

DARE – 19

CENTRAL – 931

Methods – 13

HTA - 2

NHS EED – 75

HIV PrEP

Updated Strategies

2016 Oct 7

OVID Multifile

Database: Embase <1980 to 2016 Week 40>, Epub Ahead of Print, In-Process & Other Non-Indexed Citations, Ovid MEDLINE(R) Daily and Ovid MEDLINE(R) <1946 to Present>

Search Strategy:

--------------------------------------------------------------------------------

1 HIV Seronegativity/ (45187)

2 ((AIDs or (acquired immune deficiency* adj2 virus) or (acquired immunodeficiency* adj2 virus*) or human immunodeficiency virus* or human immuno-deficiency virus*) adj2 (negativ* or seronegativ* or sero-negativ*)).tw,kw. (2330)

3 ((HIV or HIV-1 or HIV1 or HIV-I or HIVI or HIV-2 or HIV2 or HIV-2 or HIV-II or HIVII or HTLV* or LAV-2 or LAV-II) adj2 (negativ* or seronegativ* or sero-negativ*)).tw,kw. (31543)

4 ((uninfected or "not infected") adj2 (HIV or HIV-1 or HIV1 or HIV-I or HIVI or HIV-2 or HIV2 or HIV-2 or HIV-II or HIVII or HTLV* or LAV-2 or LAV-II)).tw,kw. (7561)

5 ((uninfected or "not infected") adj2 (AIDs or (acquired immune deficiency* adj2 virus) or (acquired immunodeficiency* adj2 virus*) or human immunodeficiency virus* or human immuno-deficiency virus*)).tw,kw. (330)

6 (without adj (HIV or HIV-1 or HIV1 or HIV-I or HIVI or HIV-2 or HIV2 or HIV-2 or HIV-II or HIVII or HTLV* or LAV-2 or LAV-II)).tw,kw. (3604)

7 (without adj (AIDs or (acquired immune deficiency* adj2 virus) or (acquired immunodeficiency* adj2 virus*) or human immunodeficiency virus* or human immuno-deficiency virus*)).tw,kw. (2127)

8 ((non or "not") adj (HIV or HIV-1 or HIV1 or HIV-I or HIVI or HIV-2 or HIV2 or HIV-2 or HIV-II or HIVII or HTLV* or LAV-2 or LAV-II) adj positiv*).tw,kw. (198)

9 (non-positiv* adj (HIV or HIV-1 or HIV1 or HIV-I or HIVI or HIV-2 or HIV2 or HIV-2 or HIV-II or HIVII or HTLV* or LAV-2 or LAV-II)).tw,kw. (0)

10 ((non or "not") adj (AIDs or (acquired immune deficiency* adj2 virus) or (acquired immunodeficiency* adj2 virus*) or human immunodeficiency virus* or human immuno-deficiency virus*) adj positiv*).tw,kw. (2)

11 (non-positiv* adj (AIDs or (acquired immune deficiency* adj2 virus) or (acquired immunodeficiency* adj2 virus*) or human immunodeficiency virus* or human immuno-deficiency virus*)).tw,kw. (0)

12 or/1-11 (85836)

13 exp HIV Infections/pc [Prevention & Control] (87027)

14 (prevent* adj3 (AIDs or (acquired immune deficiency* adj2 virus) or (acquired immunodeficiency* adj2 virus*) or human immunodeficiency virus* or human immuno-deficiency virus*)).tw,kw. (10628)

15 (prevent* adj3 (HIV or HIV-1 or HIV1 or HIV-I or HIVI or HIV-2 or HIV2 or HIV-2 or HIV-II or HIVII or HTLV* or LAV-2 or LAV-II)).tw,kw. (39776)

16 (prophyla* adj3 (AIDs or (acquired immune deficiency* adj2 virus) or (acquired immunodeficiency* adj2 virus*) or human immunodeficiency virus* or human immuno-deficiency virus*)).tw,kw. (721)

17 (prophyla* adj3 (HIV or HIV-1 or HIV1 or HIV-I or HIVI or HIV-2 or HIV2 or HIV-2 or HIV-II or HIVII or HTLV* or LAV-2 or LAV-II)).tw,kw. (3844)

18 or/13-17 (110965)

19 Anti-Retroviral Agents/ (41148)

20 Anti-HIV Agents/ (46834)

21 (antiretrovir* or anti-retrovir* or antiHIV* or anti-HIV* or antiAIDS or anti-AIDS).tw,kw. (142097)

22 Adenine/aa (8195)

23 tenofovir*.tw,kw. (12318)

24 107021-12-5.rn. (0)

25 (rilpivirine or edurant or TMC-278 or TMC278 or UNII-FI96A8X663).tw,kw. (1294)

26 rilpivirine.rn. (1329)

27 (dapivirine or TMC-120 or TMC120 or UNII-TCN4MG2VXS).tw,kw. (416)

28 dapivirine.rn. (62)

29 Truvada.tw,kw. (1386)

30 ("TDF-FTC" or "FTC-TDF" or "TDF/FTC" or "FTC/TDF").tw,kw. (1367)

31 731772-45-5.rn. (0)

32 or/19-31 (191205)

33 Primary Prevention/ or Pre-Exposure Prophylaxis/ (53617)

34 32 and 33 (1330)

35 12 and (18 or 34) (6345)

36 18 and 32 (20381)

37 exp HIV Infections/ (590622)

38 exp HIV/ (325246)

39 HIV Seroprevalence/ (12996)

40 (AIDs or (acquired immune deficiency* adj2 virus) or (acquired immunodeficiency* adj2 virus*) or human immunodeficiency virus* or human immuno-deficiency virus*).tw,kw. (424372)

41 (HIV or HIV-1 or HIV1 or HIV-I or HIVI or HIV-2 or HIV2 or HIV-2 or HIV-II or HIVII or HTLV* or LAV-2 or LAV-II).tw,kw. (613944)

42 or/37-41 (902547)

43 Pre-Exposure Prophylaxis/ (1509)

44 ((pre-expos* or preexpos*) adj3 prophyla*).tw,kw. (3448)

45 ((pre-expos* or preexpos*) adj3 protect*).tw,kw. (156)

46 PrEP.tw,kw. (7659)

47 or/43-46 (9722)

48 (1 or 42) and 47 (3619)

49 35 or 36 or 48 (26300)

50 Economics/ (250962)

51 exp "Costs and Cost Analysis"/ (501962)

52 Economics, Nursing/ (38713)

53 Economics, Medical/ (44510)

54 Economics, Pharmaceutical/ (10277)

55 exp Economics, Hospital/ (747372)

56 Economics, Dental/ (38244)

57 exp "Fees and Charges"/ (66902)

58 exp Budgets/ (41020)

59 budget*.ti,ab. (51589)

60 (economic* or cost or costs or costly or costing or price or prices or pricing or pharmacoeconomic* or pharmaco-economic* or expenditure or expenditures or expense or expenses or financial or finance or finances or financed).ti. (342580)

61 (economic* or cost or costs or costly or costing or price or prices or pricing or pharmacoeconomic* or pharmaco-economic* or expenditure or expenditures or expense or expenses or financial or finance or finances or financed).ab. /freq=2 (496495)

62 (cost* adj2 (effective* or utilit* or benefit* or minimi* or analy* or outcome or outcomes)).ab. (275125)

63 (value adj2 (money or monetary)).ti,ab. (4053)

64 exp models, economic/ (157949)

65 Quality-Adjusted Life Years/ (27394)

66 (life qualities or life quality or quality adjusted or adjusted life or qol or qoly or qolys or hrqol or qaly or qalys or qale or qales).tw. (140918)

67 exp Models, Economic/ (157949)

68 economic model*.ti,ab. (5792)

69 agent-based.ti,ab. (4704)

70 individual-based.ti,ab. (4851)

71 transmission dynamic?.ti,ab. (4712)

72 deterministic.ti,ab. (21942)

73 compartmental.ti,ab. (19092)

74 (compartment* adj2 model*).ti,ab. (35573)

75 (discrete event? adj2 (model* or simulat*)).ti,ab. (1478)

76 ordinary differential equation?.ti,ab. (4335)

77 ode model*.ti,ab. (451)

78 Stochastic Processes/ (13201)

79 stochastic.ti,ab. (56919)

80 markov chains/ (12108)

81 markov.ti,ab. (35588)

82 monte carlo method/ (53017)

83 monte carlo.ti,ab. (71693)

84 exp Decision Theory/ (13242)

85 (decision* adj2 (tree* or analy* or model*)).ti,ab. (38714)

86 or/50-85 (2061526)

87 49 and 86 (3496)

88 "Quality of Life"/ (504773)

89 quality-adjusted life years/ (27394)

90 (life adj1 (quality or qualities)).ti,ab. (14593)

91 (adjusted adj1 (quality or life)).ti,ab. (27717)

92 (qol or qoly or qolys or hrqol or qaly or qalys or qale or qales).ti,ab. (117566)

93 or/88-92 (553985)

94 49 and 93 (543)

95 87 or 94 (3682)

96 exp Animals/ not (exp Animals/ and Humans/) (15180232)

97 95 not 96 (2545)

98 ("20160429" or "20160430" or 201605* or 201606* or 201607* or 201608* or 201609* or 201610*).dc. (1270176)

99 97 and 98 (47)

100 99 use ppez (47)

101 ((AIDs or (acquired immune deficiency* adj2 virus) or (acquired immunodeficiency* adj2 virus*) or human immunodeficiency virus* or human immuno-deficiency virus*) adj2 (negativ* or seronegativ* or sero-negativ*)).tw,kw. (2330)

102 ((HIV or HIV-1 or HIV1 or HIV-I or HIVI or HIV-2 or HIV2 or HIV-2 or HIV-II or HIVII or HTLV* or LAV-2 or LAV-II) adj2 (negativ* or seronegativ* or sero-negativ*)).tw,kw. (31543)

103 ((uninfected or "not infected") adj2 (HIV or HIV-1 or HIV1 or HIV-I or HIVI or HIV-2 or HIV2 or HIV-2 or HIV-II or HIVII or HTLV* or LAV-2 or LAV-II)).tw,kw. (7561)

104 ((uninfected or "not infected") adj2 (AIDs or (acquired immune deficiency* adj2 virus) or (acquired immunodeficiency* adj2 virus*) or human immunodeficiency virus* or human immuno-deficiency virus*)).tw,kw. (330)

105 (without adj (HIV or HIV-1 or HIV1 or HIV-I or HIVI or HIV-2 or HIV2 or HIV-2 or HIV-II or HIVII or HTLV* or LAV-2 or LAV-II)).tw,kw. (3604)

106 (without adj (AIDs or (acquired immune deficiency* adj2 virus) or (acquired immunodeficiency* adj2 virus*) or human immunodeficiency virus* or human immuno-deficiency virus*)).tw,kw. (2127)

107 ((non or "not") adj (HIV or HIV-1 or HIV1 or HIV-I or HIVI or HIV-2 or HIV2 or HIV-2 or HIV-II or HIVII or HTLV* or LAV-2 or LAV-II) adj positiv*).tw,kw. (198)

108 (non-positiv* adj (HIV or HIV-1 or HIV1 or HIV-I or HIVI or HIV-2 or HIV2 or HIV-2 or HIV-II or HIVII or HTLV* or LAV-2 or LAV-II)).tw,kw. (0)

109 ((non or "not") adj (AIDs or (acquired immune deficiency* adj2 virus) or (acquired immunodeficiency* adj2 virus*) or human immunodeficiency virus* or human immuno-deficiency virus*) adj positiv*).tw,kw. (2)

110 (non-positiv* adj (AIDs or (acquired immune deficiency* adj2 virus) or (acquired immunodeficiency* adj2 virus*) or human immunodeficiency virus* or human immuno-deficiency virus*)).tw,kw. (0)

111 or/101-110 (44194)

112 exp Human immunodeficiency virus infection/pc [Prevention] (42208)

113 (prevent* adj3 (AIDs or (acquired immune deficiency* adj2 virus) or (acquired immunodeficiency* adj2 virus*) or human immunodeficiency virus* or human immuno-deficiency virus*)).tw,kw. (10628)

114 (prevent* adj3 (HIV or HIV-1 or HIV1 or HIV-I or HIVI or HIV-2 or HIV2 or HIV-2 or HIV-II or HIVII or HTLV* or LAV-2 or LAV-II)).tw,kw. (39776)

115 (prophyla* adj3 (AIDs or (acquired immune deficiency* adj2 virus) or (acquired immunodeficiency* adj2 virus*) or human immunodeficiency virus* or human immuno-deficiency virus*)).tw,kw. (721)

116 (prophyla* adj3 (HIV or HIV-1 or HIV1 or HIV-I or HIVI or HIV-2 or HIV2 or HIV-2 or HIV-II or HIVII or HTLV* or LAV-2 or LAV-II)).tw,kw. (3844)

117 or/112-116 (78881)

118 antiretrovirus agent/ (37328)

119 anti human immunodeficiency virus agent/ (16110)

120 (antiretrovir* or anti-retrovir* or antiHIV* or anti-HIV* or antiAIDS or anti-AIDS).tw,kw. (142097)

121 tenofovir/ (17139)

122 tenofovir.rn. (13945)

123 rilpivirine/ (1592)

124 rilpivirine.rn. (1329)

125 dapivirine/ (432)

126 dapivirine.rn. (62)

127 emtricitabine plus tenofovir disoproxil/ (2790)

128 Truvada.tw,kw. (1386)

129 ("TDF-FTC" or "FTC-TDF" or "TDF/FTC" or "FTC/TDF").tw,kw. (1367)

130 731772-45-5.rn. (0)

131 or/118-130 (174850)

132 prophylaxis/ or pre-exposure prophylaxis/ (111671)

133 131 and 132 (4197)

134 111 and (117 or 133) (4204)

135 117 and 131 (15317)

136 exp Human immunodeficiency virus infection/ (337460)

137 exp Human immunodeficiency virus/ (325246)

138 human immunodeficiency virus prevalence/ (9879)

139 (AIDs or (acquired immune deficiency* adj2 virus) or (acquired immunodeficiency* adj2 virus*) or human immunodeficiency virus* or human immuno-deficiency virus*).tw,kw. (424372)

140 (HIV or HIV-1 or HIV1 or HIV-I or HIVI or HIV-2 or HIV2 or HIV-2 or HIV-II or HIVII or HTLV* or LAV-2 or LAV-II).tw,kw. (613944)

141 or/136-140 (871301)

142 pre-exposure prophylaxis/ (1509)

143 ((pre-expos* or preexpos*) adj3 prophyla*).tw,kw. (3448)

144 ((pre-expos* or preexpos*) adj3 protect*).tw,kw. (156)

145 PrEP.tw,kw. (7659)

146 or/142-145 (9722)

147 141 and 146 (3512)

148 134 or 135 or 147 (19741)

149 economics/ (250962)

150 exp cost/ (501962)

151 exp health economics/ (725472)

152 exp fee/ (66902)

153 budget/ (38633)

154 budget*.ti,ab. (51589)

155 (economic* or cost or costs or costly or costing or price or prices or pricing or pharmacoeconomic* or pharmaco-economic* or expenditure or expenditures or expense or expenses or financial or finance or finances or financed).ti. (342580)

156 (economic* or cost or costs or costly or costing or price or prices or pricing or pharmacoeconomic* or pharmaco-economic* or expenditure or expenditures or expense or expenses or financial or finance or finances or financed).ab. /freq=2 (496495)

157 (cost* adj2 (effective* or utilit* or benefit* or minimi* or analy* or outcome or outcomes)).ab. (275125)

158 (value adj2 (money or monetary)).ti,ab. (4053)

159 statistical model/ (224516)

160 economic model*.ti,ab. (5792)

161 probability/ (124788)

162 mathematical model/ (225829)

163 agent-based.ti,ab. (4704)

164 individual-based.ti,ab. (4851)

165 transmission dynamic?.ti,ab. (4712)

166 deterministic.ti,ab. (21942)

167 compartment model/ (14194)

168 compartmental.ti,ab. (19092)

169 (compartment* adj2 model*).ti,ab. (35573)

170 (discrete event? adj2 (model* or simulat*)).ti,ab. (1478)

171 ordinary differential equation?.ti,ab. (4335)

172 ode model*.ti,ab. (451)

173 stochastic model/ (9599)

174 stochastic.ti,ab. (56919)

175 markov.ti,ab. (35588)

176 monte carlo method/ (53017)

177 monte carlo.ti,ab. (71693)

178 decision theory/ (3515)

179 (decision* adj2 (tree* or analy* or model*)).ti,ab. (38714)

180 or/149-179 (2279029)

181 148 and 180 (3116)

182 exp "quality of life"/ (527859)

183 (life adj1 (quality or qualities)).ti,ab. (14593)

184 (adjusted adj1 (quality or life)).ti,ab. (27717)

185 (qol or qoly or qolys or hrqol or qaly or qalys or qale or qales).ti,ab. (117566)

186 or/182-185 (560957)

187 148 and 186 (473)

188 181 or 187 (3305)

189 exp animal experimentation/ or exp models animal/ or exp animal experiment/ or nonhuman/ or exp vertebrate/ (43407330)

190 exp human/ or exp human experimentation/ or exp human experiment/ (34340102)

191 189 not 190 (9068859)

192 188 not 191 (3294)

193 conference abstract.pt. (2342796)

194 192 not 193 (3131)

195 ("20160429" or "20160430" or 201605* or 201606* or 201607* or 201608* or 201609* or 201610*).dd. (423562)

196 194 and 195 (46)

197 100 or 196 [BOTH DATABASES] (93)

198 remove duplicates from 197 (83) [TOTAL UNIQUE RECORDS]

199 198 use ppez [MEDLINE UNIQUE RECORDS] (46)

200 198 use emez [EMBASE UNIQUE RECORDS] (37)

***************************

**Grey literature search**

The grey literature search will consist of searching public health departments looking for PrEP economic evaluations. General search terms will be “PrEP”, “economic evaluation” “cost-effectiveness” (with and without the hyphen, “cost-utility”, “cost-benefit”. “Truvada”, “tenofovir”, “emtricitabine” are also terms that will be considered. We will identify economic evaluation studies from the websites of relevant health technology assessment (HTA) agencies. The list of these agencies from Africa, Americas, Asia, Europe, Oceania, and international organizations will be obtained from the HTAi vortal (http://vortal.htai.org/?q=organisations)., and include (but are not limited to):

- NICE (UK): <https://www.nice.org.uk/>
- CADTH (Canada): <https://www.cadth.ca/>
- EuroScan (EU): <https://www.euroscan.org/>
- University of York Centre for Reviews and Dissemination (UK): <http://www.crd.york.ac.uk/CRDWeb/>
- Cost-Effectiveness Analysis Registry (USA): <https://research.tufts-nemc.org/cear4/Home.aspx>
- HERC (USA): <http://www.herc.research.va.gov/include/page.asp?id=home>
- ISPOR: <http://www.ispor.org/>
- Cochrane Library: <http://www.cochranelibrary.com/>
- HealthEconomics.com: <http://www.healtheconomics.com/>
- Institute of Health Economics (IHE) (Canada): <http://www.ihe.ca/>
- PMREB (Canada): <http://www.pmprb-cepmb.gc.ca/en/home/>
- PHRMA: <http://www.phrma.org/>
- SMDM: <http://smdm.org/>
- CAHSPR (Canada): <http://www.cahspr.ca/>
- CHE (UK): <http://www.york.ac.uk/che/>
- CHEPA (Canada): <http://www.chepa.org/>
- International Society for Pharmacoepidemiology: <http://www.pharmacoepi.org/>
- AHRQ (USA): <http://www.ahrq.gov/>
- HTA at NIHR (UK): <http://www.nets.nihr.ac.uk/programmes/hta>
- INAHTA: <http://www.inahta.org/>
- CIHI (Canada): <https://www.cihi.ca/en>
- HAS (France): <https://www.has-sante.fr/portail/>
- IQWIG (Germany): <http://www.inahta.org/members/iqwig/>
- PBAC (Australia): <http://www.pbs.gov.au/info/industry/listing/participants/pbac>
- NZHTA (New Zealand): http://www.otago.ac.nz/christchurch/research/nzhta/
- HITAP (Thailand): <http://www.hitap.net/en/>
- NHSCRD (NHS EED) https://www.crd.york.ac.uk/CRDWeb/
