# Supplementary material for: Economic evaluation of HIV pre-exposure prophylaxis strategies: protocol for a methodological systematic review and quantitative synthesis
Source: Syst Rev. 2018 Mar 15;7:47. doi: 10.1186/s13643-018-0710-0 (PMC5855998; doi:10.1186/s13643-018-0710-0)
Supplement: Supplementary file 3 — Data abstraction form. (DOCX 41 kb) [file 13643_2018_710_MOESM3_ESM.docx]

**Additional File 3**

**Data Abstraction Form**

| **Data Extraction Variables** | **Value Type** | **Value Label** |
| --- | --- | --- |
| **Study characteristics** | | |
| What is the reference number of this article? | Integer |  |
| Covidence number | Integer |  |
| Is this a companion article?  If yes, answer questions for the primary article | Dichotomous | Yes/No |
| If this is a companion, what is the reference # of the primary article? | Integer |  |
| Last name of first author | Text |  |
| Does the economic evaluation refer to a published checklist/tool (e.g. CHEERS)? | Categorical  (Drop Down) | No |
|  |  | Yes – CHEERS |
|  |  | Yes - Phillips |
|  |  | Yes – 2^nd^ Panel on CEA in Health and Medicine |
|  |  | Yes - ISPOR |
|  |  | Yes – Other (name) |
| Other: Name of published checklist/tool | Text |  |
| Publication year | Integer |  |
| At what level was this study conducted? | Categorical  (Drop Down) | National |
|  |  | Subnational (state/province/municipal) |
|  |  | Multiple countries |
|  |  | Multiple subnational within single country |
| Location of study (countries) | Text |  |
| Location of study (subnational) | Text |  |
| Economic evaluation approach | Categorical  (Drop Down) | Patient-level (net-benefit regression) |
|  |  | Patient-level (other) |
|  |  | Decision-analytic or decision-tree |
|  |  | Hybrid (decision-tree and Markov) |
|  |  | Cohort model (Markov) |
|  |  | Microsimulation (agent-based) model without onward HIV transmission |
|  |  | Dynamic model with onward HIV transmission |
| Maximum/Ceiling Budget | Categorical  (Drop Down) | Fixed (maximum) budget |
|  |  | Comparison without fixed budget |
| If fixed budget, state budget cap | Text |  |
| **Population Characteristics & Baseline HIV Intervention Context (status quo, pre-intervention)** | | |
| Pop under study (can be >1) | Categorical  (Drop Down) | General population (not a specific key population) |
|  |  | Female - pregnant |
|  |  | Female - breastfeeding |
|  |  | MSM |
|  |  | Trans-male |
|  |  | Trans-female |
|  |  | FSW |
|  |  | Clients of FSW |
|  |  | Serodiscordant partnerships (heterosexual) |
|  |  | Serodiscordant partnerships (same sex, male) |
|  |  | Transgender male |
|  |  | Transgender female |
|  |  | Persons who inject drugs |
|  |  | Peri-conception serodiscordant partnerships (heterosexual) |
|  |  | Adolescents and young adults |
|  |  | Other: please specify |
| Pop under study: Other | Text |  |
| Population under study: additional details | Text |  |
| **Population 1 (repeat for every population from the “population under study”, and for every risk-strata within a population)** | | |
| Population size 1, % | Numeric |  |
| Population size, denominator | Categorical (Drop Down) | Adult males |
|  |  | Adult females |
|  |  | Total adults |
| Population 1 HIV prevalence (pre-intervention), % | Numeric |  |
| Population 1 HIV prevalence Year | Integer |  |
| Population 1 HIV incidence (pre-intervention) | Numeric |  |
| Population 1 HIV incidence (pre-intervention, units as per X person-years) | Integer | (e.g. 100; 100,000) |
| Does population 1 have > 1 risk-strata? | Dichotomous | Yes/No |
| How were the strata chosen within Population 1? | Categorical  (Drop Down) | Relative HIV incidence |
|  |  | Relative HIV prevalence |
|  |  | Number of sex partners |
|  |  | No details provided |
|  |  | Other: specify |
| How were the strata chosen: other | Text |  |
| **If population 1 has > 1 risk strata, repeat for each strata within each population** | | |
| Population size strata 1, % of population 1 | Numeric |  |
| Population 1, strata 1, HIV prevalence (pre-intervention), % | Numeric |  |
| Population 1, strata 1, HIV prevalence Year | Integer |  |
| Population 1, strata 1, HIV incidence (pre-intervention) | Numeric |  |
| Population 1, strata 1 HIV incidence (pre-intervention, units as per X years) | Integer | (e.g. 100; 100,000) |
| **HIV infectiousness / biological transmission probability** | | |
| HIV infectiousness units | Categorical  (Drop Down) | per sex act |
|  |  | per partnership |
|  |  | per needle-sharing episode |
| HIV infectiousness (value) - overall | Numeric |  |
| HIV infectiousness (value) - receptive anal | Numeric |  |
| HIV infectiousness (value) - insertive anal | Numeric |  |
| HIV infectiousness (value) - vaginal (male to female transmission) | Numeric |  |
| HIV infectiousness (value) - vaginal (female to male transmission) | Numeric |  |
| HIV infectiousness (value) - needle sharing | Numeric |  |
| Did HIV infectiousness vary by stage of HIV (viremia)? | Dichotomous | Yes/No |
| Reference HIV stage for relative increase in HIV infectiousness | Text | e.g. CD4 250-300; or asymptomatic stage, etc. |
| Relative increase in transmission probability relative to reference stage – acute/early HIV | Numeric |  |
| Relative increase in transmission probability relative to reference stage – late-stage HIV / AIDS | Numeric |  |
| **HIV testing (pre-intervention or among those not receiving the intervention)** | | |
| Population 1 Fraction HIV diagnosed, among persons living with HIV | Numeric |  |
| Population 1 Fraction HIV undiagnosed, among persons living with HIV | Numeric |  |
| Population 1 HIV testing: rate of HIV testing | Numeric |  |
| Population 1 HIV testing rate units (i.e. rate per X months) | Integer |  |
| HIV testing details | Text |  |
| **Antiretroviral therapy (ART, pre-intervention)** | | |
| Is ART included? | Dichotomous | Yes/No |
| If ART was included, which line of therapy was used? | Categorical  (Drop Down) | First-line regimen |
|  |  | Second-line regimen |
|  |  | Third-line regimen |
| ART – ART initiation criteria used | Categorical  (Drop Down) | Immediate |
|  |  | CD-4 criteria (list below) |
|  |  | Details not provided |
|  |  | Other (list below) |
| ART - ART initiation criteria (CD4 or other) | Text |  |
| ART - regimen (drug combination) used | Text |  |
| ART coverage (% of persons diagnosed who are on ART, pre-intervention) | Numeric |  |
| Fraction PLHIV on ART (%) | Numeric |  |
| Fraction on ART with suppressed viral load (%) | Numeric |  |
| Relative reduction in HIV infectiousness while on ART (if not virally suppressed) | Numeric |  |
| Relative reduction in HIV infectiousness while on ART (if virally suppressed) | Numeric |  |
| Mortality rate PLHIV not on ART | Numeric |  |
| Mortality rate PLHIV on ART | Numeric |  |
| Units as per X person-years for mortality rate | Integer |  |
| **Other Prevention Technologies / Practices (pre-intervention)** | | |
| Was condom use at baseline explicitly included in the model? | Dichotomous | Yes/No |
| Condom-use, per partnership or per sex act? | Categorical  (Drop Down) | per sex act |
|  |  | per partnership |
|  |  | Not specified |
| Proportion sex acts condoms used (%) | Numeric |  |
| Condom efficacy (%) | Numeric |  |
| Condom-use varied by type of sex act (anal insertive; anal receptive; vaginal)? | Dichotomous | Yes/No |
| Proportion insertive sex acts condoms used (%) | Numeric |  |
| Proportion receptive sex acts condoms used (%) | Numeric |  |
| Sero-assortive practices included? | Dichotomous | Yes/No |
| Condom-use varied by own and partner's HIV status? | Dichotomous | Yes/No |
| Was post-exposure prophylaxis included? | Dichotomous | Yes/No |
| Post-exposure prophylaxis: describe | Text |  |
| Was injecting drug use included? | Dichotomous | Yes/No |
| Proportion needles not shared (%) | Numeric |  |
| Baseline level of needle sharing/exchange: describe | Text |  |
| Baseline level of male circumcision included? | Dichotomous | Yes/No |
| Proportion of males circumcised | Numeric |  |
| Structural interventions at baseline | Dichotomous | Yes/No |
| Other interventions that interrupt HIV acquisition or transmission included? | Dichotomous | Yes/No |
| List other baseline interventions | Text |  |
| Were STI co-infection included? | Dichotomous | Yes/No |
| Baseline STI prevalence | Numeric |  |
| Which STI? | Text |  |
| Did the STI increase HIV infectiousness? | Dichotomous | Yes/No |
| Did the STI increase HIV susceptibility? | Dichotomous | Yes/No |
| **Cost** | | |
| What is the currency? | Text |  |
| What is the year of pricing? | Integer |  |
| Time horizon (years) | Integer |  |
| Cycle length (if applicable) | Integer |  |
| What is the perspective of analysis? | Categorical  (Drop Down) | Societal |
|  |  | Health-system |
|  |  | Hospital |
|  |  | Patient |
|  |  | Other (describe) |
|  |  | Not specified |
| Perspective - other | Text |  |
| How did authors describe their economic evaluation type? | Categorical (Multiple choice) | Cost-effectiveness analysis |
|  |  | Cost-utility analysis |
|  |  | Cost-benefit analysis |
|  |  | Cost-minimization |
|  |  | Other: describe |
| How did authors describe their economic evaluation type - other | Text |  |
| Were costs discounted? | Dichotomous | Yes/No |
| Annual discount rate - cost | Numeric |  |
| Were outcomes discounted? | Dichotomous | Yes/No |
| Annual discount rate - outcome | Numeric |  |
| Type of discounting: time-variant? | Dichotomous | Yes (time-variant)/No (fixed) |
| Type of discounting: equal? | Dichotomous | Yes (equal)/No (differential) |
| If discounting was differential: explain | Text |  |
| Cost estimation methods | Categorical  (Drop Down) | micro-costing |
|  |  | gross-costing |
|  |  | hybrid |
|  |  | other (describe) |
| Cost estimation method - other | Text |  |
| Type of cost included - direct, medical | Categorical  (Drop Down) | Medical - HIV tests |
|  |  | Medical - ART |
|  |  | Medical (human resources) - program, clinic (staff) |
|  |  | Medical - other services (mental health, counselling, etc.) |
|  |  | Medical - infrastructure (capital) |
|  |  | Other (list) |
| Type of cost included - direct, medical | Text |  |
| Type of cost included - direct, non-medical | Categorical  (Drop Down) | Non-medical - transportation |
|  |  | Non-medical - child care |
| Type of cost included - indirect | Categorical  (Drop Down) | Productivity loss |
|  |  | Total health-system cost (beyond individual human resources) |
|  |  | Total societal cost |
| Costs per unit - PrEP regimen (default) | Numeric |  |
| Costs per unit - PrEP regimen (lower) | Numeric |  |
| Costs per unit - PrEP regimen (upper) | Numeric |  |
| Proportion of total intervention cost due to PrEP regimen | Numeric | % |
| Was the cost of STI monitoring included? | Dichotomous | Yes/No |
| If the cost of STI monitoring was included, what was the cost for STI testing per test? | Numeric |  |
| Was the cost of HIV monitoring while on PrEP included? | Dichotomous | Yes/No |
| If the cost of HIV monitoring was included, what was the cost for HIV testing per test? | Numeric |  |
| Was the cost of other laboratory monitoring while on PrEP included? | Dichotomous | Yes/No |
| If the cost of other laboratory monitoring was included, what was the per-test cost for other laboratory testing? | Numeric |  |
| **Utility assigned to health-states (for cost-utility analysis studies)** | | |
| Type of methods used to measure health utility values | Categorical  (Drop Down) | Direct measurement: Time-trade off |
|  |  | Direct measurement: Standard gamble |
|  |  | Direct measurement: Rating scale |
|  |  | Indirect measurement: EQ-5D |
|  |  | Indirect measurement: SF-6D |
|  |  | Indirect measurement: HUI |
|  |  | Not reported |
|  |  | Others |
| Others (specify how utility values were measured). | Text |  |
| HIV uninfected (value) | Numeric |  |
| on PrEP (value) | Numeric |  |
| on PrEP (relative value) | Numeric |  |
| HIV infected (value) | Numeric |  |
| HIV infected (relative value) | Numeric |  |
| HIV infected by CD4 count or other stage of infection | Text |  |
| **Intervention (Arms)** | | |
| What type of PrEP intervention being evaluated? if select others, please describe | Text |  |
| What kind (formulation) of antiretroviral for PrEP does this study consider? | Categorical  (Drop Down) | Oral |
|  |  | Topical |
|  |  | Injectable |
|  |  | Vaginal Implant/Ring |
|  |  | Include answer = "not a specific agent" |
| Name(s) of drug/agent. Include dosing and regimen. | Text |  |
| Use of generic name or trade-name? | Categorical  (Drop Down) | Generic |
|  |  | Trade-name |
|  |  | Not applicable |
| Intermittent or continuous PrEP? | Categorical  (Drop Down) | Continuous |
|  |  | Intermittent - planned |
| Base-case / comparator - no PrEP? | Categorical  (Drop Down) | Yes - No PrEP in the base-case |
|  |  | No - PrEP included in base-case (see intervention 1) |
| Describe base-case scenario | Text |  |
| **PrEP Intervention 1 (repeat for each scenario in which any intervention or baseline parameter was varied)** | | |
| PrEP -what type of intervention? | Categorical  (drop down) | Delivery of PrEP, such as on-demand vs. infinite PrEP |
|  |  | Improving uptake of PrEP |
|  |  | Improving adherence of ART used for PrEP |
|  |  | Others |
| What type of PrEP intervention being evaluated? If select others, please describe | Text |  |
| PrEP - to whom? | Categorical  (Drop Down) | General population (not a specific key population) |
|  |  | MSM |
|  |  | FSW |
|  |  | Clients of FSW |
|  |  | Serodiscordant partnerships (heterosexual) |
|  |  | Serodiscordant partnerships (same sex, male) |
|  |  | Transgender male |
|  |  | Transgender female |
|  |  | Persons who inject drugs |
|  |  | Peri-conception serodiscordant partnerships (heterosexual) |
|  |  | Adolescents and young adults |
| PrEP - to whom (other: describe) | Text | Other: please specify |
| PrEP - to whom, risk-strata | Categorical  (Drop Down) | Highest-risk only |
|  |  | All but lowest-risk |
|  |  | All groups |
|  |  | Not applicable: no risk-stratification |
| PrEP - uptake by the intended population | Numeric |  |
| PrEP - scale-up | Categorical  (Drop Down) | Immediate |
|  |  | Gradual - define time-frame |
| PrEP - scale-up over the number of years | Numeric | Years |
| PrEP - provider (how was PrEP delivered within the health-system?) | Categorical  (Drop Down) | Provider type not included |
|  |  | Specialist Physicians |
|  |  | General Practice Physicians |
|  |  | HIV clinics |
|  |  | STI clinics |
|  |  | Public Health Clinic |
|  |  | Nurse |
|  |  | Outreach |
| PrEP - provider (how was PrEP delivered within the health-system?) - other | Text | Other: please specify |
| PrEP - adherence definition | Text |  |
| PrEP - fraction adherent | Text |  |
| Did everyone in the intended population have the same level of adherence? | Dichotomous | Yes/No |
| PrEP- How adherence was measured? | Categorical  (Drop Down) | Pill count |
|  |  | Self-reported |
|  |  | Electronic pill bottle caps |
|  |  | Laboratory tests |
|  |  | Others (please describe) |
| PrEP - adherence additional details | Text |  |
| PrEP - efficacy definition / units | Categorical  (Drop Down) | relative risk reduction in HIV acquisition per sex act (%) |
|  |  | absolute risk reduction in HIV acquisition per sex act (%) |
| PrEP - efficacy (value) | Numeric |  |
| PrEP - efficacy (upper) if range explored | Numeric |  |
| PrEP - efficacy (lower) if range explored | Numeric |  |
| Did everyone in the intended population have the same level of PrEP efficacy? | Dichotomous | Yes/No |
| PrEP - efficacy describe | Text |  |
| PrEP - efficacy and adherence correlation | Dichotomous | Yes - 1:1, No (other) |
| PrEP - other correlation between adherence and efficacy | Text |  |
| PrEP - any efficacy in reducing infectiousness if infected while on PrEP | Dichotomous | Yes/No |
| PrEP - describe any efficacy in reducing infectiousness if infected while on PrEP | Text |  |
| PrEP - duration of use: did everyone have the same duration of use? | Dichotomous | Yes/No |
| PrEP - duration of use | Categorical  (Drop Down) | Not specified |
|  |  | Lifelong |
|  |  | Fixed duration |
|  |  | Varied duration |
| PrEP - duration of use fixed |  |  |
| PrEP - duration of use fixed units | Numeric |  |
| PrEP - duration of use describe | Categorical (Drop Down) | Months, Years |
| Frequency of STI testing while on PrEP (every X months)? | Integer |  |
| Frequency of other laboratory testing while on PrEP (every X months)? | Integer |  |
| Frequency of HIV testing while on PrEP (every x months) | Integer |  |
| If one becomes infected with HIV while on PrEP - immediately diagnosed and on ART? | Categorical (Drop Down) | Yes |
|  |  | No - there is a time-lag |
| If one becomes infected with HIV while on PrEP - is there a probability of *not* getting on ART? | Dichotomous | Yes/No |
| What happens if acquire HIV while on PrEP – please describe | Text |  |
| Risk compensation by individual on PrEP included? | Dichotomous | Yes/No |
| Condomless sex increase | Dichotomous | Yes/No |
| If yes – by how much (describe)? | Text |  |
| Number of sexual partners increase | Dichotomous | Yes/No |
| If yes - by how much (describe)? | Text |  |
| Increase in number of known and disclosed status HIV+ partners | Dichotomous | Yes/No |
| If yes – by how much (describe)? | Text |  |
| Was risk compensation immediate upon starting PrEP? | Dichotomous | Yes/No |
| Risk compensation by individual on PrEP: describe | Text |  |
| Risk compensation by individuals not on PrEP included? | Dichotomous | Yes/No |
| Risk compensation by individuals not on PrEP: describe | Text |  |
| Total cost (include direct/indirect) of Intervention 1 | Numeric |  |
| Total direct health-care costs only of Intervention 1 | Numeric |  |
| Total indirect health-care costs only of Intervention 1 | Numeric |  |
| *Repeat for each PrEP Intervention* |  |  |
| **Non-PrEP, Comparator Intervention 1** | | |
| Type of non-PrEP intervention or comparator | Categorical (Drop Down) | Status-quo, ART, PEP, increasing condom use, other (describe) |
| Type of intervention - other |  |  |
| Non-PrEP intervention - to whom? | Categorical (Drop Down) | General population (not a specific key population) |
|  |  | MSM |
|  |  | FSW |
|  |  | Clients of FSW |
|  |  | Serodiscordant partnerships (heterosexual) |
|  |  | Serodiscordant partnerships (same sex, male) |
|  |  | Transgender male |
|  |  | Transgender female |
|  |  | Persons who inject drugs |
|  |  | Peri-conception serodiscordant partnerships (heterosexual) |
|  |  | Adolescents and young adults |
| Non-PrEP - to whom (other: describe) | Text | Other: please specify |
| Non-PrEP intevention coverage | Numeric |  |
| Non-PrEP intevention describe | Text |  |
| Total cost (include direct/indirect) of non-PreP Intervention 1 | Numeric |  |
| Total direct health-care costs only of non-PreP Intervention 1 | Numeric |  |
| Total indirect health-care costs only of non-PreP Intervention 1 | Numeric |  |
| *Repeat for each non-PrEP Intervention* |  |  |
| **Outcomes** | | |
| Adverse Events | | |
| Are drug-related adverse events included? | Dichotomous | Yes/No |
| List adverse events | Text |  |
| Are drug-related adverse events included in costs? | Dichotomous | Yes/No |
| Are drug-related adverse events included in outcomes (utility)? | Dichotomous | Yes/No |
| Is secondary drug resistance (by individuals on PrEP) included? | Dichotomous | Yes/No |
| Is transmitted drug resistance included? | Dichotomous | Yes/No |
| Is the type of circulating virus resistance profile included? | Dichotomous | Yes/No |
| ARV resistance - provide details |  |  |
| Health outcomes measured | | |
| Health Outcomes | Categorical (drop down) | HIV infections averted |
|  |  | HIV incidence |
|  |  | HIV prevalence |
|  |  | Lives saved |
|  |  | QALY |
|  |  | DALY |
|  |  | STI incidence: specify STI in Other |
|  |  | STI prevalence: Specify STI in Other |
|  |  | other: list |
| Health Outcomes measured - other | Text |  |
| **Effectiveness** | | |
| Were findings reported as incremental costs? | Dichotomous | Yes/No |
| Were findings reported as incremental effectiveness? | Dichotomous | Yes/No |
| **Outcome 1** | | |
| PrEP Intervention | Integer | Refers to the PrEP intervention 1, 2, 3, etc. |
| Comparator | Categorical (drop down) | Base-case |
|  |  | PrEP other: state the intervention integer |
|  |  | Non-PrEP other: state the intervention integer |
| PrEP Comparator - other | Integer |  |
| Non-PrEP comparator - other |  |  |
| Outcome 1 type | Categorical (drop down) | HIV infections averted |
|  |  | HIV incidence |
|  |  | HIV prevalence |
|  |  | Lives saved |
|  |  | QALY |
|  |  | DALY |
|  |  | STI incidence: specify STI in Other |
|  |  | STI prevalence: Specify STI in Other |
|  |  | other: list |
| Outcome 1 type - Other | Text |  |
| Cost per outcome | Numeric |  |
| Outcome per cost | Numeric |  |
| **Repeat for Outcome** |  |  |
| Willingness to pay threshold definition or source | Text |  |
| Willingness to pay threshold 1 (in USD) | Integer |  |
| Willingness to pay threshold 2 (in USD) | Integer |  |
| Willingness to pay threshold 3 (in USD) | Integer |  |
| **Sensitivity or Uncertainty Analysis** | | |
| Parameters – 1-way uncertainty | Dichotomous | Yes/No |
| Parameters - probabilistic multivariate uncertainty | Dichotomous | Yes/No |
| List intervention parameters examined in uncertainty analysis (can check more than 1) | Categorical (drop down) | PrEP efficacy |
|  |  | PrEP adherence |
|  |  | Duration on PrEP |
|  |  | Cost of PrEP as a drug |
|  |  | Frequency of HIV testing |
|  |  | PrEP coverage |
|  |  | ART uptake |
|  |  | ART coverage at baseline |
|  |  | ART efficacy at reducing HIV VL |
|  |  | other: list |
| List parameters examined in uncertainty analysis - other |  |  |
| Random variation (between agents if agent-based model) uncertainty | Dichotomous | Yes/No |
| **Reporting** | | |
| Cost-effectiveness ratios | Dichotomous | Yes/No |
| Incremental net benefit | Dichotomous | Yes/No |
| Efficiency frontier | Dichotomous | Yes/No |
| Retain dominant strategies only | Dichotomous | Yes/No |
| Value of information | Dichotomous | Yes/No |
| Budget impact | Dichotomous | Yes/No |
| **Calibration** | | |
| Was a description of the data that the model was calibrated to provided? | Dichotomous | Yes/No |
| Were details of the data that the model was fit to provided? | Dichotomous | Yes/No |
| Was the model calibrated to the HIV prevalence or incidence of more than 1 risk-group | Dichotomous | Yes/No |
| Was the model calibrated to equilibrium or trends | Dichotomous | Yes/No |
| What was the model calibration approach | Text | Target-fitting, minimize least squares, Bayesian MCMC, etc. |
| What was the model calibrated to | Text | List the data types (HIV prevalence in each group, etc.) |
| What parameters were calibrated? | Text | List the parameters that were calibrated (mixing, ART uptake, etc.) |
| **For dynamic models only** | | |
| Type of model | Categorical (Drop-down) | Deterministic compartmental |
|  |  | Stochastic compartmental |
|  |  | Network (put with individual based) |
|  |  | Individual/agent/ based or microsimulation |
|  |  | Other |
| Type of model -other | Text |  |
| Sexual mixing | Categorical (Drop-down) | Random mixing |
|  |  | Proportionate mixing |
|  |  | Between proportionate and assortative: provide level |
|  |  | Assortative |
| Level of assortative mixing | Numeric |  |
| **Other** | | |
| Conflicts of interests declared or stated none to declare for all authors | Dichotomous | Yes/No |
| At least 1 author with industry COI | Dichotomous | Yes/No |
| Was this an industry-sponsored study (even partial) | Dichotomous | Yes/No |

**Note:** HIV = human immunodeficiency virus; MSM = Men who have sex with men; FSW = Female sex workers; ART = antiretroviral treatment; STI = sexually transmitted infections; PrEP = Pre-exposure prophylaxis; HUI = health utility index; PEP =post exposure prophylaxis; QALY = quality-adjusted life year; DALY = disability-adjusted life year; MCMC = Markov chain Monte Carlo; COI = conflict of interest; PLHIV (persons living with HIV)
