# Supplementary material for: Economic evaluation of HIV pre-exposure prophylaxis strategies: protocol for a methodological systematic review and quantitative synthesis
Source: Syst Rev. 2018 Mar 15;7:47. doi: 10.1186/s13643-018-0710-0 (PMC5855998; doi:10.1186/s13643-018-0710-0)
Supplement: Supplementary file 4 — Quality assessment. (DOCX 34 kb) [file 13643_2018_710_MOESM4_ESM.docx]

**Additional File 4**

Methodological quality assessment criteria for HIV PrEP economic evaluations,

adapted from CHEERS[1], Phillips checklist[2], Second Panel on Cost-effectiveness in Health and Medicine[3], and Recommendations by model type from the International Society for Pharmacoeconomics and Outcomes Research [4-9].

Answers will be reported as:

1. Yes

2. No or not reported

3. Unclear or insufficient information to assess

| **Dimensions of quality** | **Items for critical appraisal** |
| --- | --- |
| **1. Model Structure** | |
| Statement of decision problem/objective related to PrEP | A clear statement of the decision problem related to PrEP |
|  | The objective of the evaluation and model is clearly specified and consistent with the stated decision problem |
|  | The primary decision maker for PrEP delivery is specified |
| Statement of scope/perspective | The perspective of the analysis is clearly stated |
|  | Data inputs are consistent with the stated perspective |
|  | The geographic and population scope of the model have been stated and justified |
|  | The outcomes of the model are consistent with the perspective, scope and overall objective of the model |
| Rationale for model structure (HIV-uninfected, HIV infection) | The empirical evidence regarding the model structure has been described |
|  | The structure of the model is consistent with the natural history of HIV infection |
|  | Any competing theories regarding PrEP and HIV model structure have been considered |
|  | The sources of data used to develop the structure of the model are specified |
|  | The causal relationships are described by the model structure justified appropriately |
| Structural assumptions | The structural assumptions are transparent and justified |
|  | Assumptions about sexual or injecting transmission mixing patterns are justified |
|  | The structural assumptions and simplifications are reasonable given the overall objective, perspective and scope of the model |
| PrEP strategies/comparators | There is a clear definition of the PrEP and comparator options under evaluation |
|  | All feasible and practical PrEP comparative options have been evaluated |
|  | There is justification for the exclusion of feasible PrEP or comparator options |
| Model type | The chosen model type is appropriate given the decision problem and specified causal relationships within the model |
| Time horizon | The time horizon of the model is sufficient to reflect all important *direct* differences between options |
|  | The time horizon of the model is sufficient to reflect all important *indirect* differences between options – such as prevention of onward HIV transmission or other externalities |
|  | The time horizon of the model, and the duration of treatment and treatment effect are described and justified |
|  | A lifetime horizon has been used. Alternatively, a shorter time horizon has been justified. |
| HIV infection states and HIV care pathways | The HIV states (state transition model) or the pathways (decision tree model) reflect the underlying biological process of HIV infection and the HIV care pathway and the impact of interventions. |
| Cycle length | The cycle length is defined and justified in terms of the natural history of HIV and the initiation and cessation of PrEP use. |
| **2. Data** | |
| Data identification | The data identification methods are transparent and appropriate given the objectives of the model. |
|  | Where choices have been made between data sources, these are justified appropriately. |
|  | Particular attention has been paid to identifying data for the important parameters (PrEP adherence/effectiveness, baseline HIV incidence) in the model |
|  | The process of selecting key parameters (PrEP adherence/effectiveness, baseline HIV incidence) have been justified and systematic methods used to identify the most appropriate data |
|  | The quality of the input data has been assessed appropriately, with consideration of representativeness and potential biases |
|  | Where expert opinion has been used, the methods are described and justified. |
| Pre-model data | The pre-model data analysis methodology is based on justifiable statistical and epidemiological techniques. |
| Baseline (comparator) input data | The choice of input data is described and justified. |
|  | Transition probabilities are calculated appropriately (especially if conversion from rates). |
|  | A half cycle correction has been applied to both cost and outcome? |
| PrEP effects (efficacy, effectiveness) | PrEP efficacy and effectiveness data are synthesized using appropriate techniques |
|  | The methods and assumptions used to extrapolate short-term results to final outcomes have been documented and justified. Any alternative assumptions have been explored through sensitivity analysis. |
|  | Assumptions regarding the absence or presence of a continuing effect of PrEP once PrEP is discontinued have been documented and justified. |
| Quality-of-life weights (utilities) for HIV infection | The utilities incorporated into the model are appropriate |
|  | The sources for the utility weights are referenced. |
|  | The methods of derivation for the utility weights are justified. |
|  | Utility weights are used for ‘on PrEP’ vs “HIV uninfected and not on PrEP” and if not, discussion/justification is provided. |
| Data incorporation | All data incorporated into the model have been described and referenced in sufficient details. |
|  | The use of mutually inconsistent data been is justified (i.e. are assumptions and choices appropriate). |
|  | The process of data incorporation is transparent. |
|  | If data have been incorporated as distributions, the choice of distribution for each parameter has been described and justified. |
| Assessment of uncertainty | The four principal types of uncertainty have been addressed. |
|  | If not, the omission of particular forms of uncertainty has been justified. |
|  | A value of information analysis has been conducted. |
| Methodological | Methodological uncertainties have been addressed by running alternative versions of the model with different methodological assumptions. |
| Structural | There is evidence that structural uncertainties have been addressed via sensitivity analysis. |
| Heterogeneity | Heterogeneity has been dealt with by running the model separately for different sub-groups. |
| Parameter | The methods of assessment of parameter uncertainty are appropriate. |
|  | Probabilistic sensitivity analysis for intervention parameters has been done, if not there is a justification. |
|  | If data are incorporated as point estimates, the ranges used for sensitivity analysis are stated and justified. |
| **3. Consistency** | |
| Internal consistency | There is evidence that the mathematical logic of the model has been tested thoroughly before use. |
| External consistency | The conclusions are valid given the data presented |
|  | Any counterintuitive results from the model are explained and justified. |
|  | If the model has been calibrated against independent data, any differences have been explained and justified. |
|  | The results of the model have been compared with those of previous models and any differences in results explained. |
| **4. Model Calibration** | |
| Multiple epidemic realizations have been used in the analysis. | |
| The model is calibrated or fit to observed data on HIV prevalence or incidence, and was the model calibration process clearly described | |
| The HIV transmission matrix (e.g. who has sex with whom) is calibrated to data (transmission dynamics models). | |
| The heterogeneity in HIV risk is calibrated to data. | |
| If the population size of the subgroups is important for the objective, the size estimate is justified. | |
| If the model is calibrated with multiple epidemic realizations, model outcomes are presented with their uncertainty ranges. | |

**References**

1. Husereau D, Drummond M, Petrou S, Carswell C, Moher D, Greenberg D, et al. Consolidated Health Economic Evaluation Reporting Standards (CHEERS) statement. Eur J Health Econ. 2013; 143:367-372.

2. Philips Z, Bojke L, Sculpher M, Claxton K, Golder S. Good practice guidelines for decision-analytic modelling in health technology assessment: a review and consolidation of quality assessment. Pharmacoeconomics. 2006; 244:355-371.

3. Sanders GD, Neumann PJ, Basu A, Brock DW, Feeny D, Krahn M, et al. Recommendations for conduct, methodological practices, and reporting of cost-effectiveness analyses: Second panel on cost-effectiveness in health and medicine. JAMA. 2016; 31610:1093-1103.

4. Pitman R, Fisman D, Zaric GS, Postma M, Kretzschmar M, Edmunds J, et al. Dynamic transmission modeling: a report of the ISPOR-SMDM Modeling Good Research Practices Task Force--5. Value Health. 2012; 156:828-834.

5. Roberts M, Russell LB, Paltiel AD, Chambers M, McEwan P, Krahn M. Conceptualizing a model: a report of the ISPOR-SMDM Modeling Good Research Practices Task Force--2. Value Health. 2012; 156:804-811.

6. Briggs AH, Weinstein MC, Fenwick EA, Karnon J, Sculpher MJ, Paltiel AD. Model parameter estimation and uncertainty: a report of the ISPOR-SMDM Modeling Good Research Practices Task Force--6. Value Health. 2012; 156:835-842.

7. Eddy DM, Hollingworth W, Caro JJ, Tsevat J, McDonald KM, Wong JB. Model transparency and validation: a report of the ISPOR-SMDM Modeling Good Research Practices Task Force--7. Value Health. 2012; 156:843-850.

8. Siebert U, Alagoz O, Bayoumi AM, Jahn B, Owens DK, Cohen DJ, et al. State-transition modeling: a report of the ISPOR-SMDM Modeling Good Research Practices Task Force--3. Value Health. 2012; 156:812-820.

9. Karnon J, Stahl J, Brennan A, Caro JJ, Mar J, Moller J. Modeling using discrete event simulation: a report of the ISPOR-SMDM Modeling Good Research Practices Task Force--4. Value Health. 2012; 156:821-827.
